# A preliminary metabolomics study of the database for biological samples of schizophrenia among Chinese ethnic minorities

**DOI:** 10.1186/s12888-024-05660-z

**Published:** 2024-04-09

**Authors:** Jun Ye, Haixia Chen, Yang Wang, Haini Chen, Jiang Huang, Yixia Yang, Zhen Feng, Wenfeng Li

**Affiliations:** 1 Department of Clinical Laboratory, The Second Affiliated Hospital of Guizhou Medical University, 556000 Guizhou, China; 2 Department of Clinical Biochemistry and Laboratory Medicine, Guizhou Medical University, 550001 Guizhou, China; 3 Shandong Yingsheng Biotechnology Co., Ltd., 250101 Jinan, Shandong China; 4 Department of Psychiatry, The Second Affiliated Hospital of Guizhou Medical University, Kangfu Road, 556000 Guizhou, China

**Keywords:** Schizophrenia, Ethnic genetic resources, Metabolomics, Biomarkers

## Abstract

**Background:**

Schizophrenia (SCZ) is a profound mental disorder with a multifactorial etiology, including genetics, environmental factors, and demographic influences such as ethnicity and geography. Among these, the studies of SCZ also shows racial and regional differences.

**Methods:**

We first established a database of biological samples for SCZ in China’s ethnic minorities, followed by a serum metabolomic analysis of SCZ patients from various ethnic groups within the same region using the LC-HRMS platform.

**Results:**

Analysis identified 47 metabolites associated with SCZ, with 46 showing significant differences between Miao and Han SCZ patients. These metabolites, primarily fatty acids, amino acids, benzene, and derivatives, are involved in fatty acid metabolism pathways. Notably, L-Carnitine, L-Cystine, Aspartylphenylalanine, and Methionine sulfoxide demonstrated greater diagnostic efficacy in Miao SCZ patients compared to Han SCZ patients.

**Conclusion:**

Preliminary findings suggest that there are differences in metabolic levels among SCZ patients of different ethnicities in the same region, offering insights for developing objective diagnostic or therapeutic monitoring strategies that incorporate ethnic considerations of SCZ.

**Supplementary Information:**

The online version contains supplementary material available at 10.1186/s12888-024-05660-z.

## Introduction

SCZ is a common, polygenic, and debilitating neurological disease, characterized by chronic psychotic symptoms and socio-psychological impairments [1]. its prevalence is influenced by race, nationality, and the geographical origins of immigrants and their descendants, that affecting 1% of the global population [2]. SCZ is known for its high morbidity, prolonged course, and propensity for relapse, placing an immense burden on patients, families, and society [3]. The molecular mechanisms of SCZ remain to be explore, and diagnosis relies on the subjective assessment of symptoms and medical history, including clinical questionnaires and interviews, which presents significant challenges [4]. The disorder’s etiology is believed to result from a complex interplay between genetic predispositions and environmental influences [5],with Genome-wide association studies (GWAS) identifying numerous loci significantly associated with SCZ [6]. Concordance rates in monozygotic twins are only about 50%, underscoring the role of environmental factors alongside genetics. It has been observed that both the onset and prognosis of SCZ are linked to regional and racial factors. For instance, the association of the rs1344706 single nucleotide polymorphism in *ZNF804A* gene with SCZ is verified in European populations, whereas its relevance in Asian populations should be further investigation [7]. In China, the correlation between rs1344706 and SCZ is different among provinces, suggesting regional or ethnic heterogeneity in disease susceptibility [8]. Prognostic studies across diverse regions, including various parts of Europe, Latin America, East Asia, North Africa, and the Middle East, have revealed differences outcomes of SCZ patients [9, 10]. To advance the understanding of SCZ’s genetic mechanisms, pathogenesis, and the foundations for objective diagnosis, international collaborative efforts are underway to establish psychiatric disease sample banks across different countries and ethnicities [11, 12]. The biomarkers research for SCZ is also progressing, with a focus on genes, metabolic and immune markers, brain imaging, electrophysiological traits, and integrative multi-omics and data mining approaches [13].

As an integral part of systems biology, metabolomics complements genomics, transcriptomics, and proteomics [14]. Advancements in mass spectrometry have positioned metabolomics as a powerful tool for identifying disease-related biomarkers and elucidating disease mechanisms [15]. Significant progress has been made in areas like early diagnosis, risk assessment, drug target identification, and pathophysiological research [16].

Depends on the unique regional setting of Southeast Guizhou, China—a region densely populated by ethnic minorities, with the Miao and Dong ethnicities alone comprising over 80% of the population, this study has established a database of biological samples from purebred Chinese ethnic minorities. This database serves as a resource for SCZ genetic research, contributing to the global understanding of its pathogenic roots, inheritance patterns, and genetic features. Participants from various ethnic groups in the same region have been included, and metabolomic analyses on their serum samples for SCZ have been conducted using UPLC-MS technology. This study aims to discern metabolite variances among minority ethnicities, identify disrupted metabolic pathways, investigate changes in the metabolomic profiles of SCZ among Chinese ethnic minorities, and aid in developing objective diagnostic and therapeutic monitoring strategies tailored to different ethnic groups suffering from SCZ.

## Aims and hypotheses

- We aimed to Establishment of the Biological Sample Database.
- Our hypothesis that metabolite variances among minority ethnicities.

## Materials & methods

### Study subjects

This study was conducted between 2022 and 2023 with participants from the Second Affiliated Hospital of Guizhou Medical University’s Department of Psychiatry and the Psychiatric Hospital of Qiandongnan in Guizhou Province, China. Subjects diagnosed with schizophrenia (SCZ) according to the ICD-10 criteria were selected. Informed consent was secured after selection through questionnaire surveys, and the hospital’s ethics committee approved the study. The subjects were categorized into four ethnic groups: Han, Miao, Dong, and other minorities. A team of senior psychiatrists employed the Brief Psychiatric Rating Scale (BPRS) and the Positive and Negative Syndrome Scale (PANSS) to evaluate the subjects, in addition to collecting demographic, clinical, and whole-genome DNA data. Metabolomics data comprised 30 Miao SCZ patients, 30 healthy Miao volunteers, 30 Han SCZ patients, and 30 healthy Han volunteers, all matched for age and gender. the healthy number of Dong nationality does not meet the needs of analysis, so it was not included. Serum metabolites were quantified using UPLC-MS technology to analyze ethnic differences. The inclusion criteria were: (1) ages between 15 and 65; (2) a PANSS score above 80. Exclusion criteria were: (1) schizophrenia patients with severe physical illnesses; (2) individuals with physical and laboratory findings markedly inconsistent with schizophrenia; (3) those with mental retardation, psychoactive substance-induced disorders, and organic mental disorders; (4) those with other mental conditions like depression, anxiety, or mania. Meanwhile, the criteria for including healthy controls were defined as follows: individuals aged between 15 and 65, from the same region as the schizophrenia group. Exclusion criteria included severe physical illnesses or chronic diseases, medication use within the past month, and those less than three months post-treatment completion.

### Establishment of the biological sample database

Psychiatric professionals from the afore-mentioned hospitals collected general demographic and clinical data. Fasting venous blood samples (5 ml) were taken between 7 and 9 AM, rested for 30 min, and then centrifuged at 1000 g for 10 min to separate serum samples. These samples were then aliquoted into 200 µl volumes and stored in liquid nitrogen. Genomic DNA from peripheral blood was extracted via the magnetic bead method, and upon meeting quality standards, was preserved at -80 °C, establishing the biological sample database.

### Sample processing and analysis

Prior to analysis, serum was thawed from − 80 °C in an ice bath. A 100 µl was mixed with 500 µl methanol-water solution for 15 s, then centrifuged at 4 °C and 10,000 rpm for 20 min. Afterward, 500 µl of supernatant was diluted with 250 µl ultrapure water, mixed, and centrifuged under the same conditions, the supernatant for analysis. A 10 µl sample from each was used to prepare a mixed quality control sample (QC), processed alongside the biological samples.

### Data collection

Metabolomic analysis utilized the YSLC-80 UHPLC system coupled with YS HR 1080MD mass spectrometer (Yingsheng Biotechnology, Shandong, China), in both positive and negative ion modes. The chromatography column employed Thermo Hypesil Gold column (2.1*100 mm, 1.9 μm) at 40 °C. The mobile phase consisted of Mobile phase A (0.1% formic acid for positive, 5 mM ammonium acetate for negative) and mobile phase B (methanol). The separation was conducted through the following gradient: 0—1.5 min, 98% A; 1.5—12 min, 98%– 0% A; 12—14 min, 0% A; 14—14.1 min, 0%98% A; and 14.1—17 min, 98% A. The flow rate was 0.3 mL/min. The Orbitrap MS, equipped with a heated ESI source.

The mass spectrometry parameters for positive/negative ionization modes were set as following: spray voltage 3.5Kv (+)/3.0 kV (-); temperatures of ion source, capillary, and auxiliary gas were 350 °C, 300 °C, 300 °C, respectively; the gas pressures of aux gas, sweep gas, and sheath gas were 10, 1, 40 arb, respectively. The full MS scan range was m/z 67–1000.

### Data preprocessing and metabolite identification

Raw data were processed with Compound Discoverer 3.1 software (Thermo Scientific), including baseline correction, noise filtering, spectral alignment, and peak detection.

The metabolites identified based on molecular formula matching and exact mass within a 5ppm tolerance using searches of both in-house and global databases (mzCloud, MassList, and mzVault), and a QC RSD of less than 30%. The metabolite data was then imported into SIMCA software for PCA (Principal Component Analysis) and Orthogonal Partial Least-Squares Discriminant Analysis (OPLS-DA) to visualize metabolic differences among groups. Metabolites with a Variable Importance in Projection (VIP) score > 1.0 were deemed significant for group differentiation. A permutation test with 200 iterations was performed to confirm if the constructed OPLS-DA model was valid or overfitted. Metabolites that showed significant differentiation were selected based on a combination of VIP > 1.0from the OPLS-DA model and p-values < 0.05 from Student’s t-test or Wilcoxon’s rank-sum test. The metabolites’ information was acquired from public databases, including the Human Metabolome Database (HMDB, http://www.hmdb.ca) and the KEGG database (http://www.genome.jp/kegg/pathway.html).

## Statistical analysis

Statistical analysis was conducted using SPSS 13.0, following a previously published study [17]. Demographic and clinical characteristics were assessed using the Student’s t-test, or median (interquartile range). Metabolite pathway enrichment analysis was performed using MetaboAnalyst 5.0 (https://www.metaboanalyst.ca). Additionally, the classification potential of selected metabolites was determined by calculating the area under the Receiver Operating Characteristic (ROC) curve with SPSS 13.0.

## Results

### Establishment of a biological sample database for schizophrenia among Chinese ethnic minorities

From January 2022 to June 2023, we amassed a cohort of 577 SCZ patients, capturing demographic and clinical data along with peripheral blood samples (encompassing whole-genome DNA and serum) at their initial hospital admission. The cohort consisted of 158 Han, 229 Miao, 153 Dong, and 33 patients from other ethnic minorities (Table 1). The clinical data suggested a predominance of SCZ in young to middle-aged men, often single, with lower educational attainment and higher unemployment rates, aligning with findings of a 1997 survey [18].

**Table 1:** Statistical data of SCZ patients of different ethnical

	Han	Miao	Dong	Other
N	158	229	153	37
Age (years, mea	42.88 ± 12.31	38.61 ± 13.35	40.84 ± 13.11	44.57 ± 12.70
< 30	24(15.19%)	67(29.26%)	33(21.57%)	3(8.11%)
30–39	40(25.32%)	55(24.02%)	37(24.18%)	10(27.03%)
40–49	41(25.95%)	54(23.58%)	41(26.08%)	13(35.14%)
50–59	49(31.01%)	41(17.90%)	34(22.22%)	7(18.92%)
> 60	8(2.06%)	12(5.24%)	8(5.23%)	4(10.81%)
Gender				
Male	112	142	114	23
Female	46	87	39	14
Marital status				
Single	93	129	103	16
Married	32	65	39	14
Other	33	35	11	7
Education				
Elementary	51	82	67	20
Secondary	49	75	40	9
Higher	14	22	16	1
Other	44	50	30	7
Employment				
farmer	58	114	80	26
Unemployed	90	102	60	9
Other	10	13	13	2

### Serum metabolomics analysis across ethnicities in schizophrenia patients

**Fig. 1:**
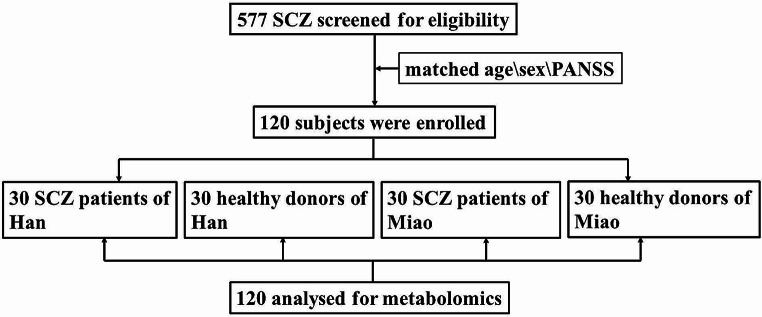
Flow chart of the study design

Utilizing the developed database, we matched 30 Han and 30 Miao SCZ patients by age, gender, and PANSS scores for our metabolomics study. Healthy Han and Miao individuals (*n* = 30 for each group) served as controls (Fig. 1). Significant disparities were observed in total protein, albumin, and indirect bilirubin among the Han SCZ patients compared to their healthy group, and in total protein, albumin and urea for the Miao SCZ patients. Furthermore, Albumin-Globulin Ratio and serum creatinine levels significantly differed between the Miao and Han schizophrenia patients (Table 2).

**Table 2:** Clinical index statistics of SCZ patients of Miao and Han

Index	MP	HP	MH	HH
gender(Male/Female)	19/11	21/9	19/11	16/14
age	40.4 ± 12.8	42.4 ± 11.9	43.6 ± 10.4	42.2 ± 11.4
PANSS	150.53 ± 19.89	151.47 ± 25.66	-	-
ALT	20.78(12.74,30.55)	14.16(10.05,30.87)	21.88(14.34,28.90)	19.73(12.98,27.1)
AST	19.60(14.50,25.78)	17.24(12.52,30.19)	18.88(15.08,24.08)	18.28(14.92,24.52)
TP	68.90(63.82,72.35)	66.73(65.22,72.45)	73.26(70.19,75.65)^#^	71.99(70.01,75.57)^&^
ALB	39.17(35.35,41.86)	40.96(39.03,43.31)	42.43(41.42,45.93)^#^	44.84(41.90,47.25)^&^
GLB	28.47(25.38,32.43)	27.56(23.95,30.70)	28.94(27.67,29.94)	27.42(26.37,29.77)
A/G	1.37(1.14,1.61)	1.43(1.17,1.78)^*^	1.52(1.45,1.62)	1.61(1.46,1.74)
TBIL	8.40(6.14,17.60)	9.11(6.76,13.71)	11.53(8.70,13.39)	11.17(9.27,13.84)
DBIL	3.07(1.92,4.73)	3.20(2.29,5.03)	2.67(2.04,3.61)	2.60(2.01,3.34)
IBIL	5.42(4.23,11.12)	6.18(3.83,8.66)	8.52(6.49,10.57)	8.76(7.39,10.37)^&^
Urea	3.16(2.86,4.06)	3.35(2.81,5.57)	4.80(4.10,5.63)^#^	4.50(3.69,5.94)
Cre	62.56(57.21,68.45)	73.82(58.01,79.56)^*^	68.97(48.77,77.68)	69.00(56.67,76.23)
Glucose	5.04(4.40,5.71)	4.65(4.10,5.18)	4.90(4.62,5.32)	4.80(4.65,5.24)

Values are presented as mean ± standard deviation, or median (interquartile range);

* represents a significance between the Miao SCZ group and the Han SCZ group(P< 0.05);

# represents a significance between the Miao SCZ group and the Miao health group(P< 0.05);

& represents a significance between Han SCZ group and Han health group(P< 0.05)

**Fig. 2:**
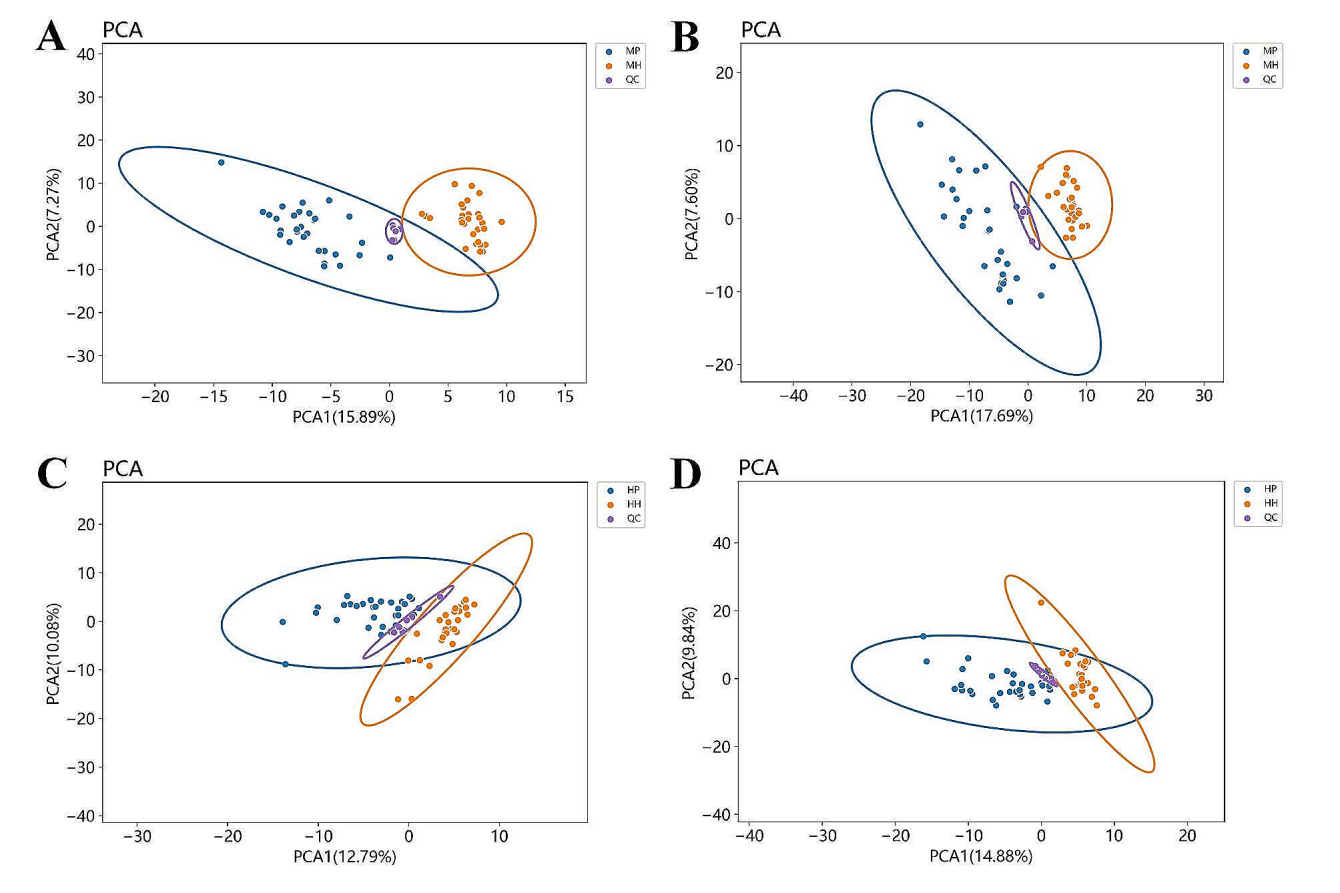
Principal Component Analysis (PCA) for Miao and Han Populations: (**A**) PCA of the positive ion mode for the SCZ and healthy groups of the Miao ethnicity; (**B**) PCA of the negative ion mode for the SCZ and healthy groups of the Miao ethnicity; (**C**) PCA of the positive ion mode for the SCZ and healthy groups of the Han ethnicity; (**D**) PCA of the negative ion mode for the SCZ and healthy groups of the Han ethnicity. QC refers to the quality control sample

**Fig. 3:**
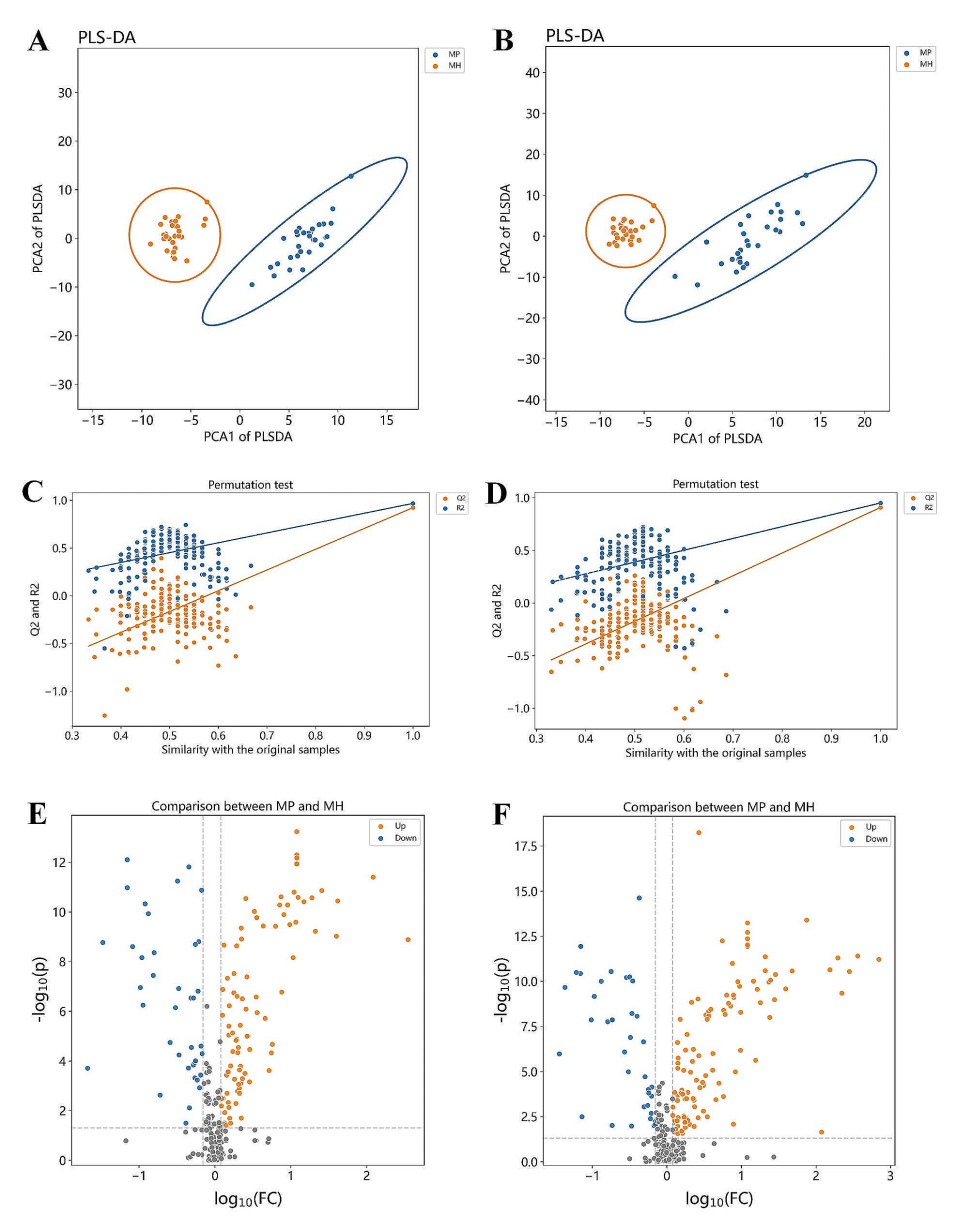
Metabolite Differentiation Screening between the Miao SCZ and Healthy Groups: (**A**) Partial Least Squares-Discriminant Analysis (PLS-DA) of the positive ion mode; (**B**) PLS-DA of the negative ion mode; (**C**) Permutation testing of the positive ion mode; (**D**) Permutation testing of the negative ion mode; (**E**) Volcano plot of the positive ion mode; (**F**) Volcano plot of the negative ion mode for the Miao schizophrenia and healthy groups

**Fig. 4:**
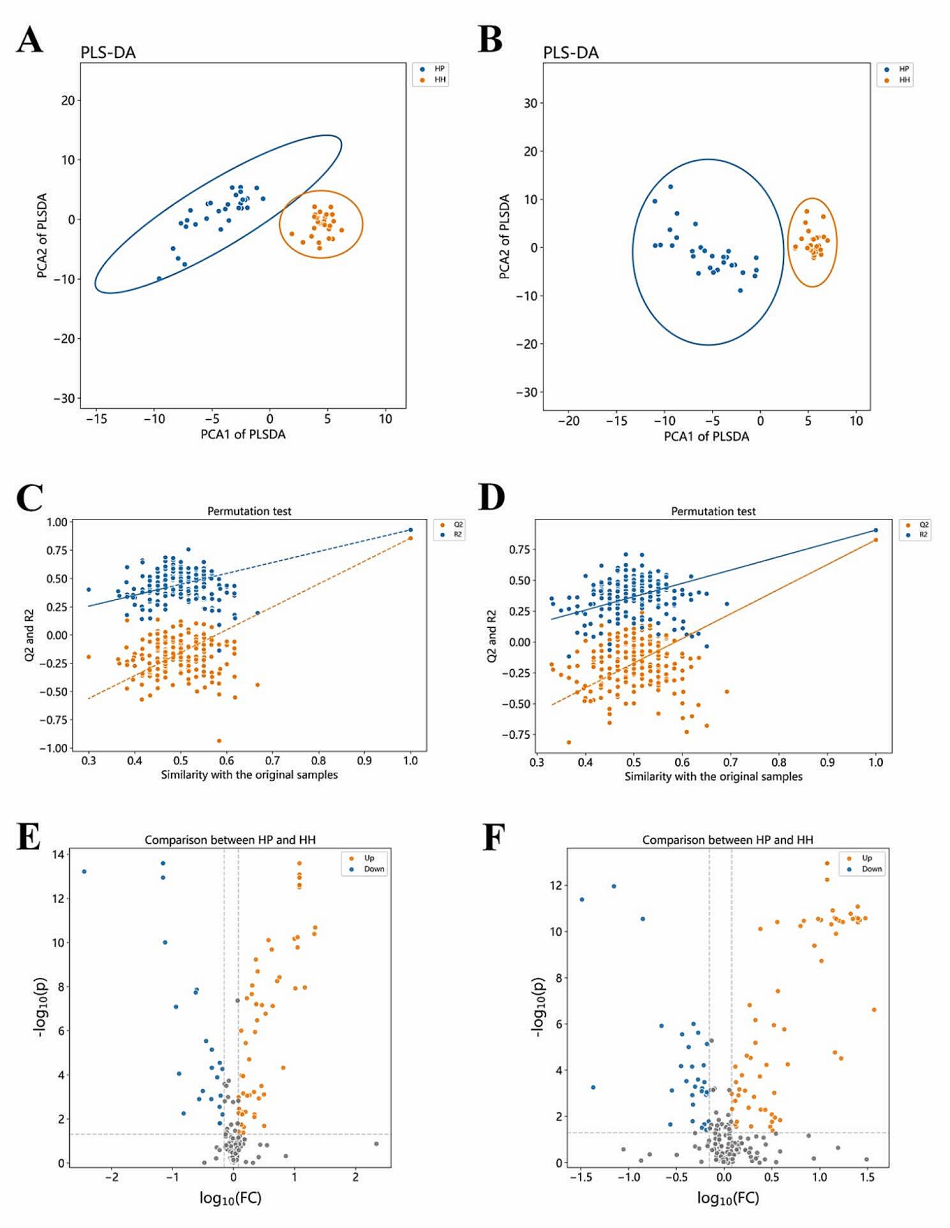
Differential Metabolite Screening between the Han SCZ and Healthy Groups: (**A**) PLS-DA of the positive ion mode for the SCZ and healthy groups; (**B**) PLS-DA of the negative ion mode for the SCZ and healthy groups of Han ethnicity; (**C**) Permutation test of the positive ion mode for the SCZ and healthy groups of Han ethnicity; (**D**) Permutation test of the negative ion mode for the SCZ and healthy groups; (**E**) Volcano plot of the positive ion mode for the SCZ and healthy groups of Han ethnicity; (**F**) Volcano plot of the negative ion mode for the SCZ and healthy groups of Han ethnicity

Based on methodical metabolomics approach (Fig. 1), the quality control samples from all groups to ensure instrumental stability, with coefficient of variation (CV) calculations assessing measurement variability. Metabolites with CVs ≤ 30% and a detection frequency over 60% were included in the analysis. PCA revealed distinct clustering of QC samples, indicated that robust sample processing and instrumental methods showed good reproducibility, and highlighted discernible metabolic profiles between the SCZ and healthy cohorts from both Han and Miao ethnicities (Fig. 2). Subsequent PLS-DA further delineated the metabolic distinctions in the Miao group (Fig. 3A and B). Permutation testing (*n* = 200) validated the PLS-DA models, confirming their reliability and absence of overfitting (Fig. 3C and D). Volcano plots illustrated that compared to healthy Miao individuals, Miao SCZ patients exhibited 104 significantly upregulated and 68 downregulated metabolites in positive ion mode, and 117 upregulated and 67 downregulated metabolites in negative ion mode. Integrating VIP scores from OPLS-DA models (VIP > 1), Student’s t-test or Wilcoxon’s rank-sum test outcomes (*p* < 0.05), and fold changes (FC < 0.7 or FC > 1.2), we identified 168 metabolites significantly altered in Miao schizophrenia patients (Fig. 3E and F). A similar analytical framework pinpointed 104 distinct metabolites in Han SCZ patients (Fig. 4A-F). Pathway enrichment analyses, a significance shifts in arachidonic acid metabolism and α-linolenic and linoleic acid pathways for the Miao, whereas Han displayed alterations in arginine and proline metabolism, followed by arachidonic acid, alanine metabolism, the urea cycle, and glycine and serine pathways (Table S1).

### Potential serum biomarkers for schizophrenia

Based on different metabolites from Han and Miao SCZ patients, we discerned 47 shared differential metabolites exhibiting consistent trends in Miao and Han group according to the FC value (Table 3). These metabolites were mainly fatty acids and derivatives (e.g., indole-3-butyric acid, 2-Oxovaleric acid, eicosapentaenoic acid), amino acids (e.g., glutamate, pyroglutamic acid, proline, taurine), and other types (e.g., bilirubin, uric acid, α-tocopherol). Pathway enrichment analysis revealed significant differences in arachidonic acid, α-linolenic and linoleic acid, and taurine and hypotaurine metabolism pathways (Table **S1**). These findings suggested that lipid and fatty acid metabolic was a common metabolic change in schizophrenia’s pathogenesis across different ethnicities.

**Table 3:** SCZ patients of Miao and Han share different metabolites

No.	HMDB	Metabolite	Class	Miao		Han	
				P value	VIP	P value	VIP
1	HMDB000	Bilirubin	Tetrapyrroles and deriv	1.20E-07	1.47	2.78E-06	1.45
2	HMDB000	Glycerophosphocholin	Glycerophospholipids	1.80E-07	1.78	3.39E-07	1.85
3	HMDB000	Glutamic acid	Carboxylic acids and d	1.23E-09	1.85	8.86E-09	2.23
4	HMDB000	Proline	Carboxylic acids and d	3.97E-06	1.44	9.85E-06	1.73
5	HMDB000	Pyroglutamic acid	Carboxylic acids and d	2.87E-11	2.33	7.76E-11	1.86
6	HMDB000	Allantoin	Azoles	3.73E-10	1.84	3.76E-09	2.11
7	HMDB000	Glutaconic acid	Carboxylic acids and d	5.23E-11	2.14	4.77E-05	1.31
8	HMDB000	Capryloylglycine	Carboxylic acids and d	1.93E-05	1.38	2.92E-04	1.17
9	HMDB000	Hypotaurine	Sulfinic acids and deriv	9.84E-05	1.24	8.78E-04	1.22
10	HMDB000	2-Aminooctanoic acid	Carboxylic acids and d	5.79E-04	1.12	4.79E-05	1.47
11	HMDB000	Angiotensin III	Carboxylic acids and d	4.34E-12	1.98	4.10E-10	1.38
12	HMDB000	Arachidonic acid	Fatty Acyls	2.60E-04	1.07	1.67E-10	2.02
13	HMDB000	20-Hydroxy-leukotrie	nFatty Acyls	1.10E-10	1.85	3.47E-11	1.83
14	HMDB000	2-Oxo-4-methylthiobu	Fatty Acyls	1.69E-10	2.04	5.77E-08	2.03
15	HMDB000	2-Oxovaleric acid	Keto acids and derivati	2.57E-11	2.13	3.16E-11	2.31
16	HMDB000	alpha-Tocopherol	Prenol lipids	3.74E-11	2.31	9.94E-11	2.43
17	HMDB000	Medroxyprogesterone	Lipids and lipid-like m	6.14E-12	2.03	3.88E-11	1.83
18	HMDB000	Eicosapentaenoic acid	Fatty Acyls	1.99E-03	1.06	6.51E-06	1.47
19	HMDB000	3-Indolebutyric acid	Indoles and derivatives	7.90E-04	1.14	1.12E-06	1.72
20	HMDB000	6-Keto-Prostaglandin	Fatty Acyls	9.85E-06	1.40	2.41E-07	1.62
21	HMDB000	Dihomo-gamma-linole	Fatty Acyls	8.58E-06	1.50	1.28E-05	1.57
22	HMDB000	Serine	Carboxylic acids and d	1.45E-04	1.03	6.92E-05	1.44
23	HMDB000	Heptan-2-one	Organooxygen compou	4.55E-10	1.06	1.13E-13	1.01
24	HMDB000	Glutamyl-taurine	Carboxylic acids and d	1.37E-11	2.22	1.15E-06	1.74
25	HMDB000	11-Dehydro-thrombox	Fatty Acyls	1.14E-12	1.64	4.08E-11	2.02
26	HMDB000	11,12-Epoxyeicosatrie	Fatty Acyls	1.14E-12	1.93	5.77E-11	2.26
27	HMDB000	8-HETE	Fatty Acyls	2.61E-10	1.71	2.59E-11	2.14
28	HMDB000	11(R)-HETE	Fatty Acyls	3.89E-12	1.81	2.87E-11	2.26
29	HMDB000	12,13-EpOME	Fatty Acyls	2.66E-11	2.09	4.75E-03	1.01
30	HMDB000	15-Deoxy-d-12,14-PG	Fatty Acyls	1.86E-10	1.62	1.23E-11	2.26
31	HMDB000	Lipoxin B4	Fatty Acyls	4.14E-11	1.92	1.26E-10	2.07
32	HMDB000	6-trans-12-epi-Leukotr	Fatty Acyls	2.28E-11	1.99	3.17E-11	2.14
33	HMDB000	20-Hydroxyeicosatetra	Fatty Acyls	2.87E-11	2.14	2.87E-11	2.34
34	HMDB000	Mevalonolactone	Lactones	5.77E-11	2.24	9.90E-07	1.68
35	HMDB000	9-cis-Retinal	Prenol lipids	1.14E-12	2.21	2.09E-11	2.37
36	HMDB001	5,15-DiHETE	Fatty Acyls	9.91E-09	1.30	2.66E-11	2.10
37	HMDB001	gamma-Glutamylgluta	Carboxylic acids and d	2.32E-04	1.14	1.69E-07	1.82
38	HMDB001	18R-HEPE	Fatty Acyls	4.94E-12	1.94	1.70E-11	2.17
39	HMDB001	Monobutylphthalate	Benzene and substitute	2.87E-11	1.84	2.87E-11	1.93
40	HMDB003	Lysopine	Carboxylic acids and d	1.27E-10	1.96	2.05E-10	1.72
41	HMDB004	3,4-Dimethoxyphenyl	eBenzene and substitute	1.29E-07	1.46	1.17E-03	1.06
42	HMDB005	Pantolactone	Lactones	2.39E-15	2.09	6.06E-05	1.43
43	HMDB024	4-Hydroxybenzophen	oBenzene and substitute	6.51E-06	1.33	3.04E-02	1.15
44	HMDB024	1,5-Anhydro-d-mannit	Organooxygen compou	2.30E-09	1.82	6.10E-03	1.09
45	HMDB024	(9Z,11E,13S,15Z)-13-	Fatty Acyls	1.04E-10	1.92	5.77E-11	2.13
46	HMDB024	4-Oxoproline	Carboxylic acids and d	5.70E-19	2.30	3.88E-11	1.88
47	HMDB025	Isophthalamide	Benzene and substitute	3.24E-11	1.84	4.16E-12	1.89

### Analysis of potential serum biomarkers for schizophrenia in Chinese ethnic minorities

**Fig. 5:**
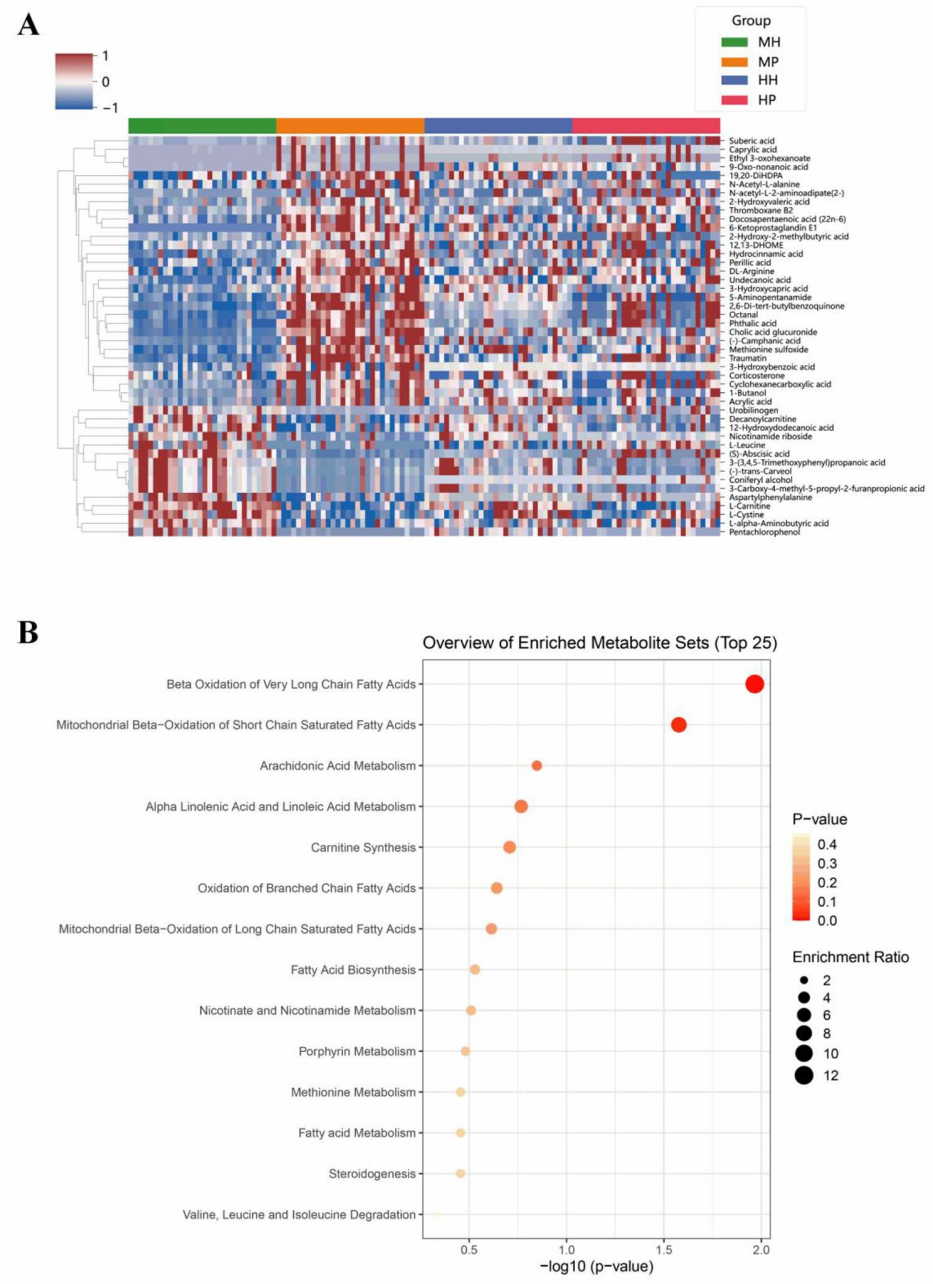
Differential Metabolite Analysis: (**A**) Heat map of differential metabolites; (**B**) Pathway analysis diagram of differential metabolites

Two metabolite datasets of Han and Miao were analyzed as a whole, 46 metabolites uniquely altered in Miao SCZ patients, that significantly differed between Miao patients and healthy Miao controls but not between Han patients (Table 4). HMDB database information has been verified, these metabolites predominantly were fatty acids (such as caprylic acid, octanedioic acid), amino acids (like arginine, leucine), and phenolics (like 3-hydroxybenzoic acid). The heatmap (Fig. 5A) showed the relative abundance shifts of these 46 metabolites among the groups. Pathway analysis implicated their involvement in processes like very long-chain fatty acids β-oxidation and mitochondrial short-chain fatty acid β-oxidation (Fig. 5B).

**Table 4:** Differential metabolites unique to Miao SCZ patients

No.	Metabolite	HMDB	*p*	VIP	FC	Class
1	Phthalic acid	HMDB0002107	3.64E-10	2.04	4.33	Benzene and substituted derivatives
2	3-Hydroxybenzoic acid	HMDB000	0.000173	1.34	1.67	Benzene and substituted derivatives
3	Methionine sulfoxide	HMDB000	1.69E-10	2.04	3.56	Carboxylic acids and derivatives
4	Cystine	HMDB000	1.55E-09	1.77	0.61	Carboxylic acids and derivatives
5	Arginine	HMDB025	2.14E-09	1.76	1.32	Carboxylic acids and derivatives
6	N-acetyl-L-2-aminoadipa	HMDB006	6.83E-09	1.39	10.77	Carboxylic acids and derivatives
7	5-Aminopentanamide	HMDB001	4.13E-08	1.83	2.65	Carboxylic acids and derivatives
8	Aspartylphenylalanine	HMDB000	2.90E-07	1.58	0.52	Carboxylic acids and derivatives
9	Leucine	HMDB000	7.12E-07	1.03	0.30	Carboxylic acids and derivatives
10	Aminobutyric acid	HMDB000	2.51E-05	1.11	0.66	Carboxylic acids and derivatives
11	N-Acetyl-L-alanine	HMDB000	0.00094	1.07	1.21	Carboxylic acids and derivatives
12	Acrylic acid	HMDB003	0.000163	1.33	2.17	Carboxylic acids and derivatives
13	Cyclohexanecarboxylic a	HMDB003	2.86E-05	1.34	2.03	Carboxylic acids and derivatives
14	Decanoylcarnitine	HMDB000	0.000145	1.12	0.52	Fatty Acyls
15	Traumatin	HMDB003	9.97E-12	2.06	7.56	Fatty Acyls
16	Undecanoic acid	HMDB000	5.85E-10	1.75	6.34	Fatty Acyls
17	2-Hydroxy-2-methylbutyr	HMDB000	9.29E-10	1.31	2.60	Fatty Acyls
18	9-Oxo-nonanoic acid	HMDB009	9.39E-10	1.14	39.81	Fatty Acyls
19	Suberic acid	HMDB000	5.18E-09	1.16	9.78	Fatty Acyls
20	3-Carboxy-4-methyl-5-pr	HMDB006	1.37E-08	1.37	0.19	Fatty Acyls
21	Docosapentaenoic acid (2	HMDB000	1.12E-06	1.45	3.61	Fatty Acyls
22	2-Hydroxyvaleric acid	HMDB000	0.000388	1.08	2.31	Fatty Acyls
23	Caprylic acid	HMDB000	0.022798	1.08	118.87	Fatty Acyls
24	6-Ketoprostaglandin E1	HMDB000	1.14E-12	1.92	12.00	Fatty Acyls
25	Thromboxane B2	HMDB000	1.01E-06	1.22	4.13	Fatty Acyls
26	19,20-DiHDPA	HMDB001	0.000335	1.15	1.76	Fatty Acyls
27	12,13-DHOME	HMDB000	0.010993	1.03	2.32	Fatty Acyls
28	12-Hydroxydodecanoic a	HMDB000	0.000101	1.20	0.56	Hydroxy acids and derivatives
29	3-Hydroxycapric acid	HMDB000	0.000145	1.29	1.91	Hydroxy acids and derivatives
30	Ethyl 3-oxohexanoate	HMDB003	5.05E-13	1.09	12.00	Keto acids and derivatives
31	Camphanic acid	HMDB034	1.05E-05	1.79	8.29	Lactones
32	Carnitine	HMDB000	1.33E-11	1.91	0.67	Organonitrogen compounds
33	1-Butanol	HMDB000	5.79E-05	1.36	3.11	Organooxygen compounds
34	Nicotinamide riboside	HMDB000	8.17E-07	1.10	0.27	Organooxygen compounds
35	Octanal	HMDB000	2.32E-09	1.93	7.15	Organooxygen compounds
36	Pentachlorophenol	HMDB004	1.05E-06	1.56	0.04	Phenols
37	Coniferyl alcohol	HMDB001	1.37E-08	1.37	0.19	Phenols
38	3-(3,4,5-Trimethoxyphen	HMDB003	4.37E-09	1.40	0.16	Phenylpropanoic acids
39	Hydrocinnamic acid	HMDB000	5.82E-07	1.21	2.26	Phenylpropanoic acids
40	Abscisic acid	HMDB003	2.49E-09	1.90	0.08	Prenol lipids
41	2,6-Di-tert-butylbenzoqui	HMDB001	5.77E-13	2.12	5.54	Prenol lipids
42	Carveol	HMDB000	1.75E-08	1.32	0.16	Prenol lipids
43	Perillic acid	HMDB000	1.33E-06	1.41	2.68	Prenol lipids
44	Cholic acid glucuronide	HMDB000	5.23E-11	2.04	7.15	Steroids and steroid derivatives
45	Corticosterone	HMDB000	0.000403	1.13	1.39	Steroids and steroid derivatives
46	Urobilinogen	HMDB000	1.35E-08	1.07	0.10	Tetrapyrroles and derivatives

**Fig. 6:**
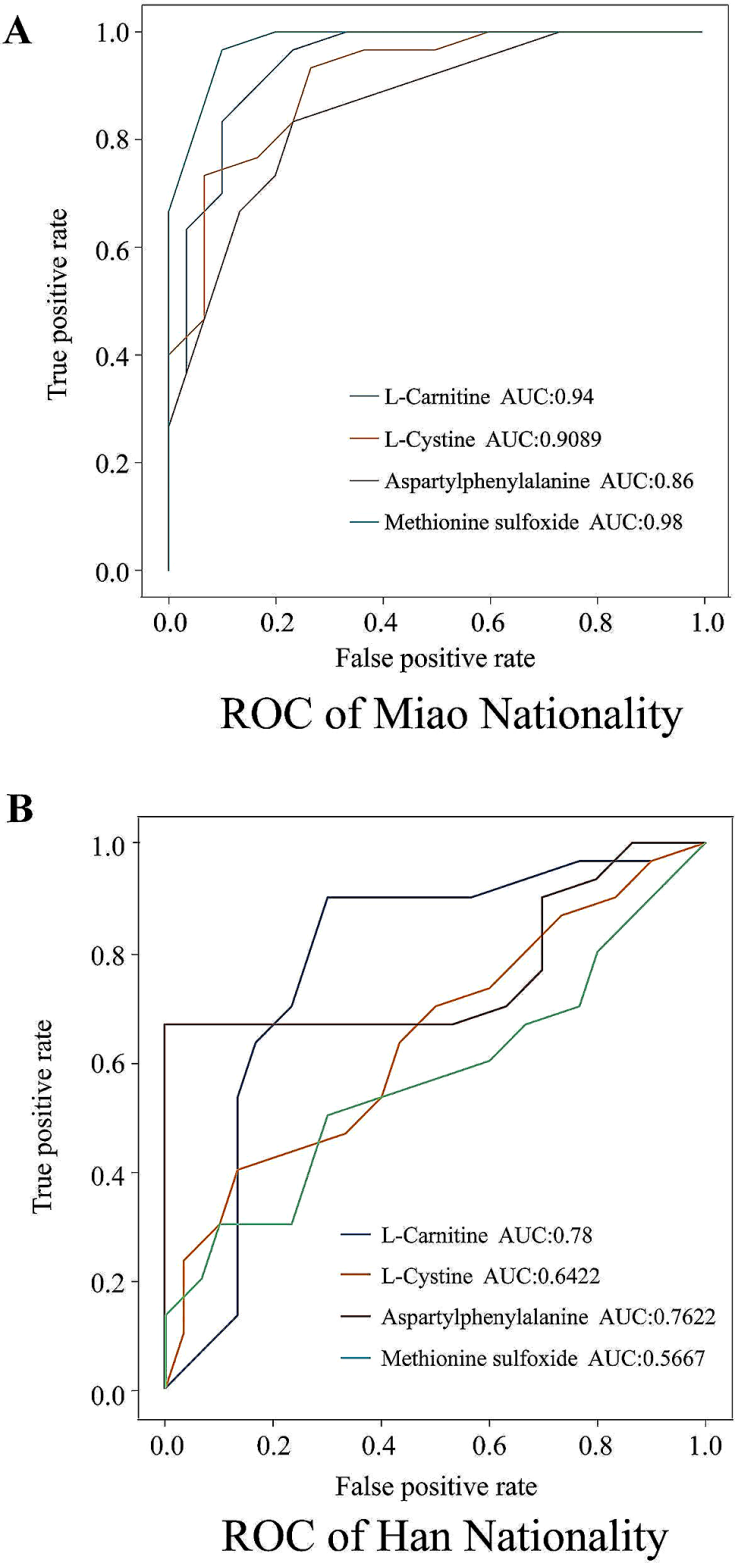
Potential Diagnostic Biomarkers: (**A**) ROC curve analysis of potential biomarkers in the Miao population; (**B**) ROC curve analysis of potential biomarkers in the Han population

To assess the identified metabolites’ potential as diagnostic or therapeutic predictors for SCZ across ethnicities, we constructed a logistic regression model and performed ROC analysis. The ROC of L-Carnitine, L-Cystine, Aspartylphenylalanine, and Methionine sulfoxide between Miao and Han groups were notable different, with respective AUC values of 0.94, 0.9089, 0.86, 0.98 for Miao, and 0.78, 0.6422, 0.7622, 0.5667 for Han (Fig. 6A-B). These markers may hold clinical value for monitoring and evaluating SCZ treatment efficacy across different ethnicities.

## Discussion

Extensive literature suggests that SCZ is a complex polygenic disorder, with its etiology and pathogenesis still not completely understood, thus representing a persistent research challenge. Consequently, acquiring pure biological samples is crucial for SCZ research to augment the global biological resources on ethnic variations in SCZ. Through in-depth exploration within isolated minority ethnic communities in China, we have assessed SCZ patients using established schizophrenia rating scales. We have compiled their clinical data, genomic DNA, and serum samples, and conducted multifactorial correlation analyses. Our preliminary results indicate differences in clinical indicators among SCZ patients across Southeast Guizhou’s ethnic minorities (Table Table 2), affirming the distinct ethnic and regional disparities in SCZ.

Given the etiological heterogeneity and absence of reliable molecular diagnostic tools, SCZ diagnosis currently hinges on the subjective recognition of clinical symptoms. This underscores the pressing need for new molecular biomarkers to enhance SCZ diagnosis and treatment. Omics techniques, such as transcriptomics and proteomics, are increasingly applied to identify biomarkers that used to reflect disease processes or response to therapeutic interventions. Metabolomics, a branch dedicated to the analysis of small-molecule metabolites in biological samples, is integral to modern systems biology. It posits that metabolites underpin phenotypic-traits, thus significantly differing metabolites (end-products of biological processes) may provide more objective and reliable disease diagnosis and prognosis. Our investigation, utilizing the SCZ biological sample database from Chinese ethnic minorities, conducted a thorough LC-MS-based metabolomics analysis on serum samples from various ethnic groups. Our goal was to identify potential ethnic-specific biomarkers or combinations thereof for SCZ, aiming to establish objective diagnostic or therapeutic monitoring strategies. Multivariate and univariate statistical analyses 47 differential metabolites in both Miao and Han SCZ patients. Research of SCZ had shown that metabolites were related to SCZ like free fatty acids, lipids, and amino acids. This study detected metabolites from these categories, and some findings align with those reported in existing research, namely Proline, Serine, Glutamic acid, Allantoin, and Glycerophosphocholine [19]. However, there were also inconsistencies [20]. Bilirubin, a metabolic byproduct formed during the conversion of hemoglobin to heme in red blood cells, is processed in the liver by UDP-glucuronosyltransferase (UGT). Elevated levels of bilirubin in the central nervous system can lead to transient bilirubin encephalopathy. Moreover, bilirubin acts as a potent endogenous antioxidant in plasma, capable of combating oxidative stress, which is implicated in the pathophysiology of SCZ. Studies have indicated that SCZ patients often exhibit reduced levels of bilirubin [21, 22]. Correspondingly, this study observed bilirubin irregularities in patients with SCZ from both the Miao and Han Chinese populations, with a general trend of reduction when compared to healthy controls.

Indole-3-butyric acid, a tryptophan metabolite dependent on gut microbiota, has also been implicated in SCZ pathogenesis through gut-brain interactions affecting brain function and behavior [23]. Patients with SCZ exhibit elevated levels of 3-indoleacetic acid in their plasma, a finding that is corroborated by this study [24]. Although changes in tryptophan levels were not observed here, these results point to the potential of exploring the interplay between SCZ, tryptophan, and the gut microbiota as a promising area for future research. Moreover, this study identified certain metabolites that are oxidized lipids, including 20-Hydroxy-leukotriene B4, 6-Keto-prostaglandin F1a, 11-Dehydro-thromboxane B2, and 8-HETE. These compounds arise from the lipid peroxidation of polyunsaturated fatty acids, phospholipids, and cholesterol esters in cell membranes and lipoproteins, a process triggered by free radicals under conditions of oxidative stress. Oxidized lipids identified in our study emerge under oxidative stress and were significant in SCZ, pointing to the need for further investigation into their roles in the disease [25, 26].

An intriguing aspect of our findings is the identification of 46 significantly differential metabolites in Miao SCZ patients, pointing towards a distinct metabolic alteration within this ethnic group. Pathway enrichment analysis indicated these metabolites primarily engage in fatty acid metabolism pathways, suggesting a more extensive alteration of metabolite levels in these pathways in Miao SCZ. This study observed significant variations in certain metabolites previously reported in Miao patients with schizophrenia (SCZ), unlike in Han patients. Notable among these were Aspartylphenylalanine, Decanoylcarnitine, 2-Hydroxyvaleric acid, and 12-Hydroxydodecanoic acid [20]. A regression model identified four metabolites—L-Carnitine, L-Cystine, Aspartylphenylalanine, and Methionine sulfoxide—as having superior diagnostic efficacy for SCZ in the Miao population compared to the Han population (Fig. 6A-B). Carnitine, a key regulator of lipid metabolism, facilitates the transport of long-chain fatty acids into the mitochondrial matrix for energy production via β-oxidation and the citric acid cycle. Additionally, carnitine is essential for removing excess acyl groups, maintaining intracellular coenzyme A balance, antioxidation, and modulating cholinergic neurotransmission [27]. First-episode schizophrenia patients exhibit increased levels of long-chain acylcarnitines and decreased levels of short-chain acylcarnitines [28]. Correspondingly, decreased levels of Carnitine and Decanoylcarnitine were noted in Miao SCZ patients in this study.

Yang et al. indicates that SCZ patients have elevated cysteine levels in their urine but reduced levels in serum compared to healthy controls [29]. The cystine/glutamate antiporter, responsible for the reciprocal transport of cystine and glutamate across the cell membrane, plays a role in this balance. Cystine, a non-essential amino acid and a precursor for glutathione synthesis, is involved in regulating the intracellular redox balance, while glutamate is an essential amino acid. Imbalances in the cystine/glutamate ratio can lead to physiological disorders [30]. This study also found elevated glutamate levels in both Miao and Han SCZ patients [31], suggesting a more significant change in the cystine/glutamate ratio among the Miao population [32]. This raises the question of whether there is an ethnic difference in the expression of the cystine/glutamate antiporter, warranting further exploration.

Methionine can be oxidized by reactive oxygen species to form two diastereoisomers of Methionine sulfoxide (MetO): methionine-S-sulfoxide (Met-S–(O)) and methionine-R-sulfoxide (Met-R–(O)), in a process involving methionine-S-sulfoxide reductase A (MsrA) and B (MsrB) enzymes. Mutations in the *MSRA* gene have been linked to an increased risk of developing SCZ and various behavioral phenotypes [33]. This study found increased expression of Methionine sulfoxide in Miao SCZ patients, indicating that further research is needed to understand the role of *MSRA* transcription and Methionine sulfoxide levels in the antioxidative mechanisms in SCZ.

However, this study is not without limitations, including a small sample size and the exclusion of certain ethnic groups due to incomplete clinical scales, highlighting the need for validation in larger, more diverse cohorts.

## Conclusion

Our findings highlight a significant association between schizophrenia and metabolic levels in lipid metabolism and redox processes, with more pronounced changes in Miao patients, pointing to ethnic variations in the metabolic profile of schizophrenia.

### Electronic supplementary material

Below is the link to the electronic supplementary material.

Supplementary Material 1

## Data Availability

All data following URL:https://www.ncbi.nlm.nih.gov/geo/query/acc.cgi?acc=GSE85337.

## Abbreviations

SCZ: Schizophrenia
GWAS: Genome-wide association studies
BPRS: Brief Psychiatric Rating Scale
PANSS: Positive and Negative Syndrome Scale
OPLS-DA: Orthogonal Partial Least-Squares Discriminant Analysis
VIP Variable: Importance in Projection
HMDB: Human Metabolome Database
ROC: Operating Characteristic
UGT: UDP-glucuronosyltransferase
MP: Miao patient
HP: Han patient
MH: Miao health
HH: Han health
ALT: Alanine aminotransferase
AST: Aspartate aminotransferase
TP: Total Protein
ALB: Albumin
GLB: Globulin
A/G: Albumin /Globulin
TBIL: total bilirubin
DBIL: Direct Bilirubin
IBIL: Indirect Bilirubin
Cre: creatinine
